# Arsenic sulfide induces RAG1-dependent DNA damage for cell killing by inhibiting NFATc3 in gastric cancer cells

**DOI:** 10.1186/s13046-019-1471-x

**Published:** 2019-12-10

**Authors:** Ting Kang, Maolin Ge, Ruiheng Wang, Zhen Tan, Xiuli Zhang, Chuanying Zhu, Han Liu, Siyu Chen

**Affiliations:** 1 Department of Oncology, Xin Hua Hospital, School of Medicine, Shanghai Jiao Tong University, Shanghai, 200092 China; 2 State Key Laboratory of Medical Genomics, Shanghai Institute of Hematology, Rui Jin Hospital, School of Medicine and School of Life Sciences and Biotechnology, Shanghai Jiao Tong University, Shanghai, 200025 China

**Keywords:** Arsenic sulfide, Gastric cancer, NFATc3, DNA damage, RAG1

## Abstract

**Background:**

Arsenic sulfide was found to have potential anti-cancer activities, especially in gastric cancer. However, the underlying mechanism need to be further explored. This study was aimed to investigate the mechanism of arsenic compounds on gastric cancer.

**Methods:**

Gastric cancer cell lines were infected with lentiviral vector carrying shNFATc3 and/or treated with arsenic sulfide. MTT assay were performed to assess cell growth. Flow cytometer assays were used to detect cell cycle and reactive oxygen species (ROS) level of gastric cancer cells. Western blot was carried out to detect nuclear factor of activated T-cells, cytoplasmic 3 (NFATc3), cell cycle markers, DNA damage pathway protein expression as well as other protein expression in gastric cancer cell lines. The expression of recombination activating gene 1 (RAG1) in gastric cancer cell lines was determined by RNA-sequencing analyses and Real-Time qPCR. The effect of NFATc3 on RAG1 were determined by CHIP-qPCR assay. The effect of arsenic sulfide on AGS cells was evaluated in vivo.

**Results:**

We show that arsenic sulfide as well as knockdown of NFATc3 resulted in increased double-strand DNA damage in gastric cancer cells by increasing the expression of RAG1, an endonuclease essential for immunoglobulin V(D) J recombination. Overexpression of NFATc3 blocked the expression of RAG1 expression and DNA damage induced by arsenic sulfide. Arsenic sulfide induced cellular oxidative stress to redistribute NFATc3, thereby inhibiting its transcriptional function, which can be reversed by N-acetyl-L-cysteine (NAC). We show that NFATc3 targets the promoter of RAG1 for transcriptional inhibition. We further showed that NFATc3 upregulation and RAG1 downregulation significantly associated with poor prognosis in patients with gastric cancer. Our in vivo experiments further confirmed that arsenic sulfide exerted cytotoxic activity against gastric cancer cells through inhibiting NFATc3 to activate RAG1 pathway.

**Conclusion:**

These results demonstrate that arsenic sulfide targets NFATc3 to induce double strand DNA break (DSB) for cell killing through activating RAG1 expression. Our results link arsenic compound to the regulation of DNA damage control and RAG1 expression as a mechanism for its cytotoxic effect.

## Background

Gastric cancer remains a leading cause of cancer mortality worldwide and represents a significant public health burden [1]. For early stage disease, surgery plus pre-operative or post-operative chemotherapy remains the best curative treatment strategy. But, many patients have inoperable disease at diagnosis or have recurrent disease after resection with curative intent. Unfortunately, conventional chemotherapy has shown limited efficacy, with median overall survival of only 10 months [2]. Treatment options after failure of standard first-line platinum and fluoropyrimidine-based combination therapy are scarce [3], therefore identifying rational targets for developing new treatments is urgently needed.

Arsenic compounds have shown cytotoxic activities in solid tumors including gastric cancer. In acute promyelocytic leukemia, it targets promyelocytic leukemia protein (PML) for degradation of the oncogenic fusion protein PML-RARα resulted from the (15;17) gene translocation [4]. The complete remission rate of arsenical (arsenic trioxide or arsenic sulfide)-based therapy have reached a high level in patients with acute promyelocytic leukemia, making it become the first cured leukemia [5–7]. In solid tumors, studies have shown that arsenic compounds including arsenic sulfide can directly or indirectly target several signal transduction and apoptotic pathways. Previous studies suggested that arsenic trioxide could eliminate latent cancer cells in the bone marrow to prevent metastasis and reduce the risk of recurrence [8]. We and other researchers have reported that arsenic compounds inhibited the migration and invasion of gastric cancer and other solid tumor cells [9, 10]. So, the inhibiting effect of arsenic compounds on solid tumor cells has been well demonstrated. But, the exact mechanisms underlying its efficacies have not been fully understood.

Nuclear factor of activated T cells (NFAT) was first identified more than two decades ago as an inducible DNA-binding factor that binds to the interleukin-2 promoter in activated T cells [11, 12]. The NFAT family consists of five members: NFATc1, NFATc2, NFATc3, NFATc4 and NFAT5 (also known as tonicity enhancer binding protein) [13–15]. NFAT1–NFAT4 proteins are dephosphorylated by activated calcineurin, which leads to their nuclear translocation and induction of NFAT-mediated gene transcription or inhibition. The calcineurin inhibitors cyclosporine A (CsA) and FK506 prevent this dephosphorylation and NFAT nuclear accumulation [14, 16]. Calcineurin is an enzyme that has been shown to be sensitive to ROS [17]. Increased expression of NFAT proteins has been shown in many human solid tumors and hematologic malignancies, in particular, the activation of NFATc3 has been associated with poor prognosis in patients with colorectal cancer [18]. Previously, we found that NFATc3 expression was obviously higher in gastric cancer tissues compared with adjacent normal tissues [19]. Thus, it seems worthwhile to study the role of NFAT in gastric cancer biology more detailed.

Here we show that NFATc3 expression is critically important for growth and survival of gastric cancer. Mechanistically, arsenic sulfide treatment of gastric cancer cells as well as knockdown of NFATc3 induce double strand DNA break and RAG1 expression. Forced expression of NFATc3 blocked the stimulation of DSB and RAG1 expression. NFATc3 targets the promoter of RAG1 for transcriptional inhibition. Our results link arsenic compound to the regulation of DNA damage control and RAG1 expression as a mechanism for its cytotoxic effect.

## Materials and methods

### Cell culture and reagent

AGS and MGC803 were purchased from the Cell Bank, Chinese Academy of Sciences (Shanghai, People’s Republic of China), MKN45 were kindly provided by Institute of Digestive of Shanghai Ruijin Hospital affiliated with Shanghai Jiao Tong University and grown as recommended. Experiments were performed on cell lines cultured for less than 30 passages. Mycoplasma was tested monthly following an established procedure. Highly purified As4S4 was supplied by the Shanghai Institute of Hematology (Shanghai, People’s Republic of China) and was prepared as previously described [20]. Cyclosporin A and Z-VAD-FMK were obtained from Selleck. N-acetyl-L-cysteine was purchased from Sigma Aldrich (Sangon Biotech, People’s Republic of China).

### Cell proliferation, cell-cycle analysis

CellTiter 96® AQueous Non-Radioactive Cell Proliferation Assay was performed in 96-multiwell plates. Cells were incubated with 0.5 mg/mL MTS (Sigma Aldrich) at 37 °C for 2 h; then, absorbance was measured at 490 nm. Cell-cycle analyses were performed by Click-iT® Plus EdU Flow Cytometry Assay Kits (Cat. no. C10633, life) according to the manuscript.

### Western blotting

Protein lysates, loaded at equal quantity, were separated on a NuPAGE Novex 10% Bis-Tris Protein Gel (Thermo Fisher Scientific), followed by iBlot transfer to PVDF (Thermo Fisher Scientific). Membranes were blocked in 5% skim milk powder in PBS with 0.1% Tween-20, and then probed with the following antibodies, as indicated in the figure legends: polyclonal rabbit anti-NFATc3 (Ab6666, ABclonal), monoclonal mouse anti-β-actin (A1978, Sigma-Aldrich), monoclonal rabbit anti-MDM2 (D1V2Z) (86,934, CST), monoclonal mouse anti-p53(DO-1) (554,293, BD), monoclonal mouse anti-p21(554,262, BD), monoclonal rabbit anti-ATM (Phospho-Ser1981)(D6H9) (5883, CST), monoclonal rabbit anti-ATR (Phospho-Ser428) (2853, CST), monoclonal rabbit anti-CHK2 (2662, CST), monoclonal rabbit anti-p-CHK2 (Phospho-Thr68) (2661, CST), monoclonal mouse anti-CHK1(2G1D5) (2360, CST), monoclonal rabbit anti-p-CHK1(Phospho-Ser345), monoclonal rabbit anti-phosphorylated histone H2AX(Phospho-Ser139) (9718, CST), monoclonal mouse anti-RAG1(D-5)(sc-377,127, Santa Cruz), followed by goat anti–rabbit IgG antibodies conjugated to HRP or goat anti–mouse IgG antibodies conjugated to HRP, respectively (Southern Biotech).

### Quantitative reverse-transcription PCR

RNA was isolated with Spin Column Animal Total RNA Purification Kit (Sangon Biotech) and reverse-transcribed with the High-Capacity cDNA Reverse Transcription Kit (Thermo Fisher Scientific). cDNA was amplified using SYBR-Green PCR Master Mix (Thermo Fisher Scientific). When possible, primers were designed to span exon-exon junctions. Gene expression fold changes were normalized to GAPDH. Primer sequences are listed in Additional file 1: Table S1.

### ROS detection

Intracellular ROS measurements were performed using Reactive Oxygen Species Assay Kit (S0033, Beyotime). Briefly, cells were harvested and resuspended in 100 μL medium containing 10 μM DCFH-DA. Cells were then incubated for 30 min at 37 °C and used for analysis by flow cytometry.

### Lentiviral vectors

Target sequence (cgtctcagttacaacctatta and ccagatgattgtgcatccat) against human NFATc3 and target sequence (gtgagggaaatgagtctggta and gcaaagaggttccgctatgat) against human RAG1 were inserted into the pLKO.1-TRC vector, according to the manufacturer’s protocol (Addgene). For exogenous expression of NFATc3, the full-length cDNA corresponding to NFATc3 transcript variant 1 (NM_173165.2) was inserted into the pCDH-MSC-T2A-copGFP-MSCV (hereafter pCDH) (System Biosciences) according to the manufacturer’s protocol. The RAG1 cDNA (NM_001760.4) was inserted into the pCDH lentiviral vector. Lentiviral particles were produced by calcium phosphate transfection of plasmid vectors into HEK-293 T cells in combination with helper plasmids. Twenty-four hours later, the supernatant was collected, 0.45 mm filtered, and used to transduce cells by spinoculation in the presence of 8 mg/mL polybrene (Sigma-Aldrich). Transduced cells were either FACS-purified on the basis of the fluorescent reporter protein or selected with 1μg/mL puromycin (Sigma-Aldrich).

### Chromatin Immunoprecipitation

1 × 10^7^ cells were used per ChIP assay according to a published protocol [21]. Briefly, cells were crosslinked with 1% paraformaldehyde for 15 min and were quenched with glycine for 5 min at room temperature. Fixed chromatin was sonicated with a Covaris Focused-ultra sonicator and immunoprecipitated with the NFATc3 (Ab6666, ABclonal) antibody. ChIP-qPCR was performed using SYBR-Green PCR Master Mix (Thermo Fisher Scientific) on a ViiA7 PCR machine (Applied Biosystems). Relative enrichments are presented as percentage input. Primer sequences are available on Additional file 1: Figure S6.

### In vivo xenograft experiments

Twelve non-obese diabetic/severe combined immunodeficient (NOD/SCID) mice (5–7 weeks old, Beijing Vital River Laboratory, Beijing, China) were divided into 2 groups with 6 mice in each group. The mice were provided with sterile feeding and drinking water with alternated day and night for 12 h. A total of 5 × 10^6^ AGS single cells suspended in 200 μl of solution (50% PBS and 50% Matrigel) were subcutaneously inoculated into the right flank of the mice using 1 mL syringes. The mice (6 in each group) were intraperitoneally injected respectively with vehicle or 2 mg/kg arsenic sulfide. The long diameter (a) and short diameter (b) of tumors were measured every 2 days using the calipers. The tumor volume was estimated using the following formula: V (mm3) = [ab^2^]/2. Tumor tissue was removed from the tumor-bearing mice following the final treatment. All animal procedures were conducted according to protocols approved by the Institutional Animal Care and Ethics Committee of Shanghai Jiao Tong University.

### Patients and tissue samples

The gastric cancer samples and their corresponding adjacent normal tissues were obtained from 6 patients diagnosed with gastric cancer and treated with surgery at the Xinhua Hospital affiliated to Shanghai Jiao Tong University School of Medicine. All samples and clinical information were obtained with informed consent from the patient or their family. Tissues were immediately snap-frozen in liquid nitrogen after surgery and stored at − 80 °C until RNA extraction. This study was approved by the ethics committee of Xinhua Hospital affiliated to Shanghai Jiao Tong University School of Medicine.

### Statistical analysis

The Student’s t-test was used to analyze the differences between the groups. A *p* value less than 0.05 was considered to be statistically significant. (*p < 0.05, **p < 0.01, ***p < 0.001).

## Results

### NFATc3 is required for the proliferation of gastric cancer cells

**Fig. 1:**
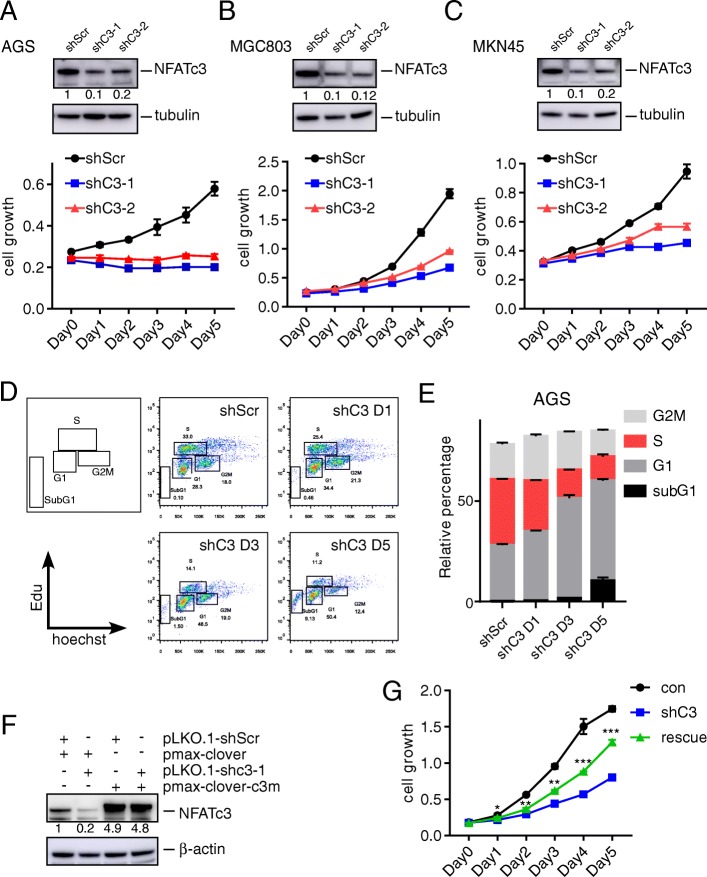
NFATc3 silencing inhibits viability and proliferation of gastric cancer cell line. **a-c** Immunoblot and the cell growth of AGS (**a**), MGC803 (**b**), and MKN45 (**c**) cells infected by the indicated lentiviral vectors. **d, e** Cell cycle profiling of the indicated AGS cells (**d**). Stacked barplot shows the fraction of cells viable in G1, S and G2/M phases of the indicated cells (**e**) (The percentage of cell population at each phases are represented as mean ± S.D. of three independent experiments). **f** NFATc3 expression in shC3–1 and the derivative line expressing the shC3–1- resistant NFATc3. Fold changes relative to first line are indicated. **g** The proliferation of the indicated AGS cells for 6 days. Statistical significance was assessed using two-way ANOVA. *p < 0.05; **p < 0.01; ***p < 0.001 versus shC3

To investigate if NFATc3 is required for the growth of gastric cancer cells, we knocked down NFATc3 in three human gastric cancer cell lines: AGS, MGC803 and MKN45. We used RFP-expressing pLKO.1-shNFATc3 shRNA constructs (shC3–1 and shC3–2) and pLKO.1-TRC-shScrambl lentiviral vector as control. As shown in Fig. 1a–c, both shC3–1 and shC3–2 effectively knocked down the expression of NFATc3 in all three cell lines and resulted in significant inhibition of cell growth.

We analyzed the effect of NFATc3 knockdown on the cell cycle regulation. The vector carrying shC3–1 was selected for the subsequent experiments due to its consistent knockdown effect. As shown in Fig. 1d–e, NFATc3 knockdown for 1 to 5 days decreased the proportion of cells in the S phase but increased the proportion of cells in the G0–G1 phase and Sub-G1phase in a time-dependent fashion, consistent with the results from Fig. 1a–c. Similar results were observed in the MGC803 and MKN45 cells (Additional file 1: Figure S1a–S1d). These findings were not due to off-target effects, because the expression of an NFATc3 cDNA carrying synonymous point mutations on the shRNA target sequence (NFATc3-mut; Additional file 1: Figure S1e) partially rescued the proliferation of cells impaired by NFATc3 knockdown (Fig. 1f, g). These results indicate that NFATc3 is required for the growth of gastric cancer cells.

### NFATc3 knockdown induces DNA double-strand breaks

**Fig. 2:**
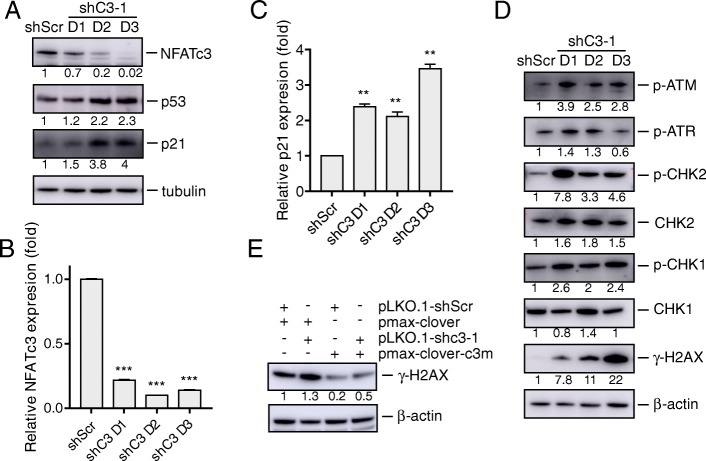
NFATc3 silencing upregulates DNA damage related genes. **a** AGS cells were infected by the indicated lentiviral vectors analyzed after infection day1 to day3. Immunoblot of the indicated antibodies were analyzed. Fold changes relative to shScr are indicated. **b, c** mRNA levels analyzed by qRT-PCR of NFATc3 (**b**) and p21 (**c**) in indicated AGS cells after infection day1 to day3. Statistical significance was assessed using two-tailed Student’s t-test. **p < 0.01; ***p < 0.001. **d** Immunoblot of DNA damage related gene set expression in that lentivirus shC3–1 or shScr infected cells. Fold changes relative to shScr are indicated. **(e)** γ-H2AX expression in shC3–1 and the derivative line expressing the shC3–1-resistant NFATc3 cDNA (pmax-clover-C3m). Fold changes relative to first line are indicated

We investigated the effect of NFATc3 on cell cycle regulators and the DNA damage pathways. We found that NFATc3 knockdown upregulated the protein levels of p53 and p21 (Fig. 2a). qRT-PCR analysis validated the effect of NFATc3 silencing and confirmed the increased level of p21 mRNA (Fig. 2b, c, Additional file 1: Figure S2a, S2b). By inducing cell cycle arrest in the pre-DNA synthesis phase, we hypothesized that NFATc3 silencing may cause significant DNA damage in the gastric cancer cells. To test our hypothesis, we measured phosphorylated ATM and phosphorylated ATR expression, which are sensors and transducers of DNA damage [22]. In NFATc3-silenced AGS cells, expression of phosphorylated ATM was markedly upregulated while the levels of phosphorylated ATR were moderately upregulated (Fig. 2d). The downstream proteins of ATM and ATR, including CHK1 and CHK2 and their phosphorylated forms, were upregulated as well (Fig. 2d). In addition, we found that phosphorylated histone H2AX (γ-H2AX) which reflects the presence of DNA double-stranded breaks was significantly increased by the NFATc3 knockdown (Fig. 2d). These effects were also observed in MGC803 cells (Additional file 1: Figure S2c). The increased level of γ-H2AX was time-dependent indicating that NFATc3 knockdown did in fact cause DSB in gastric cancer cells. In addiction, the expression of NFATc3-mut reversed the effect of NFATc3 knockdown on the stimulation of γ-H2AX (Fig. 2e). These data indicate that NFATc3 knockdown causes DSB in gastric cancer cells by activating the ATM-CHK2-p53-p21 pathway and that activation of DSB may be a mechanism responsible for the cell death resulting from the inhibition of NFATc3.

### Arsenic causes DNA damage by inhibiting NFATc3

**Fig. 3:**
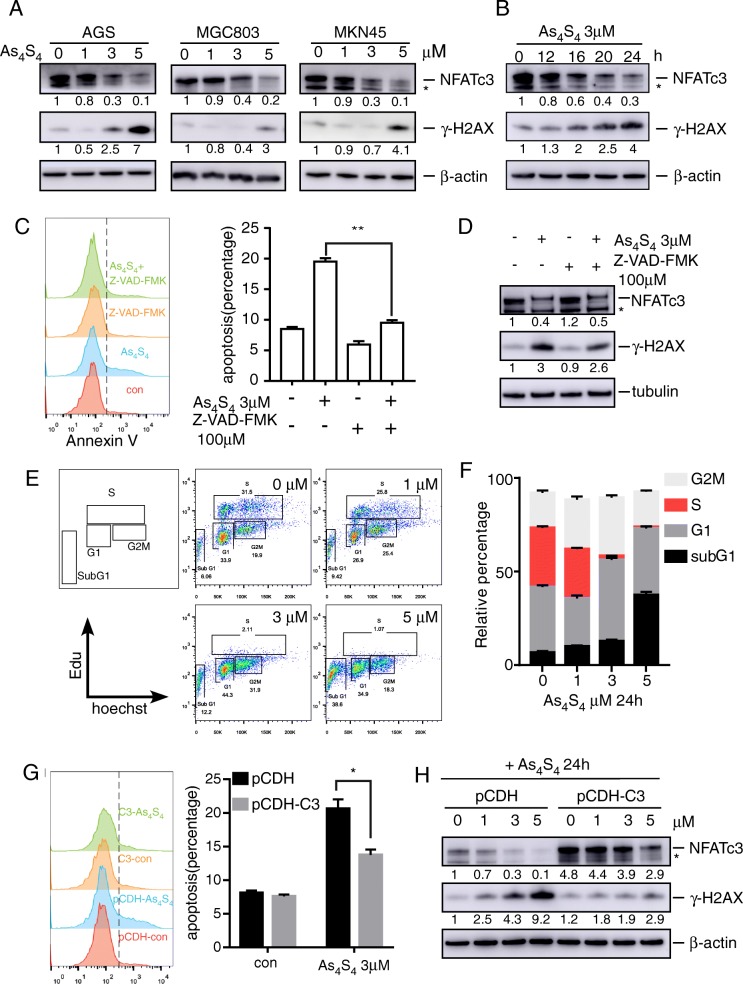
Arsenic sulfide suppresses NFATc3 to induce DNA damage. **a** Immunoblot analysis of NFATc3 and γ-H2AX expression in arsenic sulfide treated AGS, MGC803 and MKN45 cells. Fold changes relative to 0 μM are indicated. **b** Immunoblot analysis of NFATc3 and γ-H2AX expression in arsenic sulfide treated AGS cells. Fold changes relative to 0 h are indicated. **c, d** AGS cells pretreated with the pancaspase inhibitor Z-VAD-fmk (100 μM) for 1 h, then treated with arsenic sulfide for further 24 h, the viability was analyzed using flow cytometry assay (**c**) and Immunoblot (**d**). **e, f** Cell cycle of AGS cells infected by the indicated lentiviral vectors (**e**). Stacked barplot shows the fraction of cells viable in G1, S and G2/M phases of the indicated cells (**f**) (The percentage of cell population at G1, S, and G2/M phases are represented as mean ± S.D. of three independent experiments). **g** Flow cytometry analysis of apotosis in arsenic sulfide treated the indicated AGS cells. **h** Immunoblot analysis of NFATc3 and γ-H2AX expression in arsenic sulfide treated the indicated AGS cells. Fold changes relative to first line are indicated

Our previous study showed that the expression levels of NFATc3 correlated with the sensitivity of gastric cancer cells to arsenic sulfide-induced cytotoxicity [19]. We investigated if arsenic sulfide inhibits gastric cancer by targeting NFATc3 to increase DSB. We treated AGS cells with different concentrations of arsenic sulfide for 24 h. Arsenic sulfide indeed increased the level of γ-H2AX in a dose-dependent (Fig. 3a) and time-dependent manner (Fig. 3b). Similar results were obtained with MGC803 and MKN45 cells (Fig. 3a). By qRT-PCR we confirmed the effect of arsenic sulfide inhibition on NFATc3 mRNA expression (Additional file 1: Figure S3a, b).

It is well known that extensive DSBs occur during apoptosis due to caspase-activated DNase, inducing very high levels of γ-H2AX [23, 24]. To confirm the contribution of caspases in arsenic sulfide-induced DSB, we conducted an inhibitory assay with the pan-caspase inhibitor Z-VAD-FMK. Indeed, Z-VAD-FMK partly restored cell viability (Fig. 3c) and damped the upregulation of γ-H2AX (Fig. 3d), suggesting that caspase-dependent apoptosis plays a role in arsenic sulfide-induced DSB. Arsenic sulfide arrested cells in the G0–G1 phase and reduced the number of cells entering S phase (Fig. 3e, f). Similar results were obtained with MKN45 cells (Additional file 1: Figure S3c, 3d).

To verify that the inhibitory effect of arsenic sulfide on gastric cancer cells was meditated by NFATc3, we established an NFATc3-overexpressing cell line and treated it with different concentrations of arsenic sulfide. We found that overexpression of NFATc3 partly restored cell viability of AGS cells treated with 3 μM arsenic sulfide (Fig. 3g) and blocked the stimulation of arsenic sulfide on the level of γ-H2AX (Fig. 3h). This indicates that NFATc3 was responsible for the increased level of γ-H2AX upon arsenic sulfide treatment of gastric cancer cells and demonstrates that arsenic sulfide induces DNA damage by inhibiting the NFATc3 pathway.

### Arsenic sulfide increases cellular ROS to alter NFATc3 localization

**Fig. 4:**
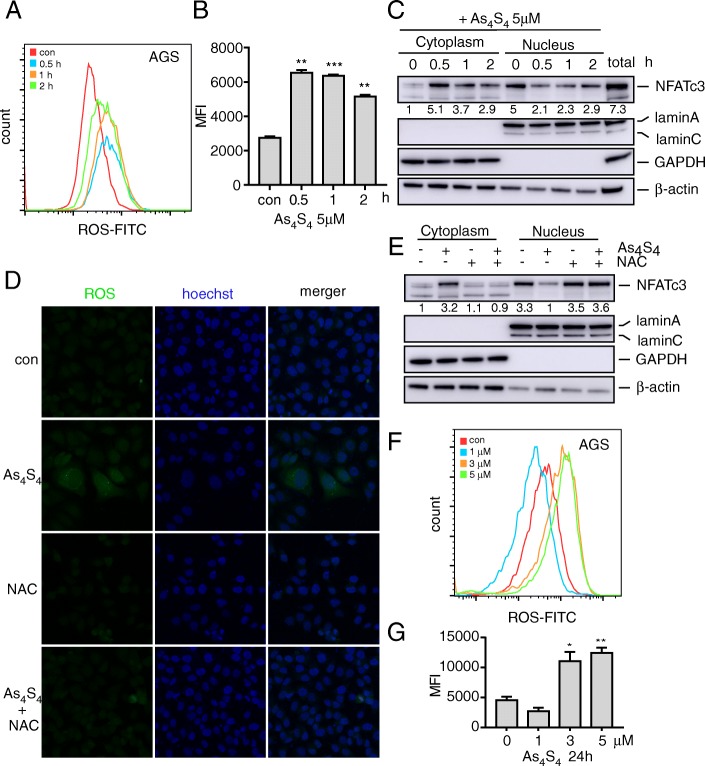
Arsenic sulfide increases celluar ROS and NFATc3 re-localization. **a, b** AGS treated with or without arsenic sulfide were stained with DCFH-DA (10 μM, 20 min, 37 °C) (**a**) and mean fluorescence intensity (MFI) of DCFH-DA was analyzed in each cell subset (**b**). Statistical significance was assessed using two-tailed Student’s t-test. **p < 0.01; ***p < 0.001. **c** The distribution of NFATc3 in cytoplasm and nucleus after arsenic sulfide (5 μM) treatment. Fold changes of NFATc3 protein relative to first line are indicated. **d, e** AGS treated with contral, arsenic sulfide (5 μM), NAC (10 mM) and arsenic sulfide plus NAC were stained with DCFH-DA (10 μM, 20 min, 37 °C) and used for ROS detection by microscopy. NAC treatment was given 1 h prior to arsenic sulfide exposure. Cells were counterstained using Hoechst (**d**). The distribution of NFATc3 in cytoplasm and nucleus after treated. Fold changes of NFATc3 protein relative to con are indicated (**e**)**.** Fold changes of NFATc3 protein relative to first line are indicated. **f, g** AGS treated with or without arsenic sulfide were stained with DCFH-DA (10 μM, 20 min, 37 °C) (**f**) and MFI of DCFH-DA was analyzed in each cell subset (**g**). Statistical significance was assessed using two-tailed Student’s t-test. *p < 0.05; **p < 0.01

We have previously shown that arsenic sulfide inhibits NFATc3 at the transcriptional level and degrades its protein. Other studies have shown that arsenic trioxide binds vicinal cysteines and increases ROS production [25]. To investigate whether ROS levels were enhanced because of arsenic sulfide treatment, DCFH-DA on a fluorescence microplate was used to test the presence of ROS. As expected, AGS cells treated with arsenic sulfide exhibited a dramatic increase in the DCFH-DA fluorescent signal compared to the control (Fig. 4a, b). We found that ROS levels peaked at 0.5 h after start of the arsenic sulfide treatment, which could be compensated by intracellular redox systems that temporarily reduced ROS levels at 1 and 2 h. Similar results were observed in two other gastric cancer cell lines (Additional file 1: Figure S4a, b).

Calcineurin contains iron and zinc at its active site that are both very sensitive to high ROS levels. To investigate whether higher levels of ROS caused by arsenic sulfide treatment alters NFATc3 localization in cancer cells, we treated cells with 1 μM cyclosporine A (CsA, as a positive control) or 5 μM arsenic sulfide for 0, 0.5, 1 and 2 h. We found that over 70% of NFATc3 localized to the nucleus of untreated AGS cells (Fig. 4c, Additional file 1: Figure S4c), indicating constitutive NFAT activation under the resting conditions in these cells. After the treatment with CsA, we observed NFAT translocation to the cytoplasm (Additional file 1: Figure S4c). Moreover, western blot analysis showed that the cells exhibited less NFATc3 nuclear accumulation after exposure to arsenic sulfide compared to the controls (Fig. 4c). Interestingly, the western blot results were consistent with the results obtained by flow cytometry. The distribution of NFATc3 in the nucleus after arsenic sulfide treatment was lowest at 0.5 h, with partial recovery within 1–2 h. ROS production was significantly abolished by incubation with 10 mM freshly prepared NAC (pH 7.4) for 1 h prior to arsenic sulfide treatment (Fig. 4d). Western blot results showed that NAC treatment also attenuated arsenic-induced NFATc3-cytoplasmic localization (Fig. 4e), indicating ROS was upstream of NFATc3 nuclear-cytoplasmic shuttling. NAC significantly reduced the levels of cell death, suggesting that arsenic-induced cell death was ROS-dependent (Additional file 1: Figure S4d). The 24-h effect showed that ROS levels gradually increased with increasing drug concentration (Fig. 4f, g). However, compared with the control group, the intracellular ROS level in the arsenic sulfide treatment group at 1 μM was decreased. These experiments confirmed that arsenic sulfide increases cellular ROS which altered the cytoplasmic localization of NFATc3. As NFATc3 is known to be regulated by nuclear-cytoplasmic shuttling, cytoplasmic localization may affect the transcriptional function of NFATc3.

### NFATc3 silencing and arsenic sulfide treatment upregulate RAG1

**Fig. 5:**
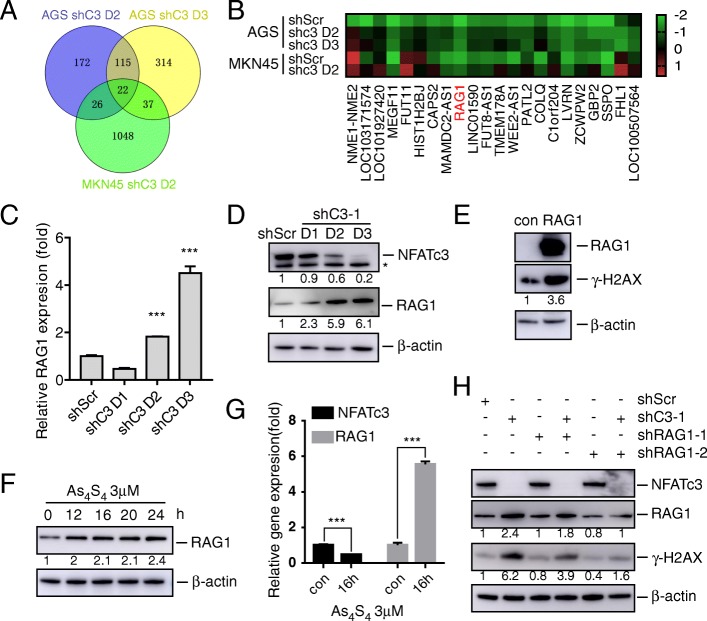
NFATc3 silencing and arsenic sulfide treatment upregulate RAG1. **a** The Venn diagram displays overlaps among LogFC ≥2 genes in response to shC3 treatment in the AGS-shC3 day2 (blue), AGS-shC3 day3 (orange) and MKN45-shC3 day2 (green). **b** Heatmap of 22 genes significantly modulated in indicated cell lines. **c** qRT-PCR analysis of RAG1 expression in lentivirus shC3–1 or shScr infected AGS cells for the indicated time points. Statistical significance was assessed using two-tailed Student’s t-test. ***P < 0.001. **d** Immunoblot analysis of RAG1 expression in lentivirus shC3–1 or shScr infected AGS cells for the indicated time points. Fold changes relative to shScr are indicated. **e** Immunoblot analysis of RAG1 and γ-H2AX expression in RAG1-overexpressed 293 T cells. Fold changes of γ-H2AX protein relative to con are indicated. **f** Immunoblot analysis of RAG1 expression in arsenic sulfide treated AGS cells. Fold changes relative to first line are indicated. **g** qRT-PCR analysis of RAG1 expression in arsenic sulfide treated AGS cells. Statistical significance was assessed using two-tailed Student’s t-test. ***p < 0.001. **h** Immunoblot analysis of γ-H2AX expression in AGS cells which RAG1 and shC3–1 both knockdown. Fold changes relative to first line are indicated

To investigate the mechanisms responsible for the NFATc3 dependence by the gastric cancer cells, we performed RNA-seq analysis of NFATc3-silenced AGS and MKN45 cells and found that 22 genes were differentially expressed in both cell lines (Fig. 5a). As a first step toward functional annotation, we examined published literature for the association of these 22 genes with the keywords “*DNA damage*” in the PubMed database. This analysis identified four genes (RAG1, CAPS2, HIST1H2BJ and FHL1) that were associated with DNA damage (Fig. 5b). Surprisingly, the search terms *(DNA damage) AND RAG1* produced 81 best-matched results. We confirmed the stimulation of RAG1 caused by NFATc3 knockdown with RT-PCR (Fig. 5c, Additional file 1: Figure S5a) and western blots (Fig. 5d). To investigate whether upregulation of RAG1 caused DSBs, we constructed a RAG1-overexpression recombination plasmid. We found that RAG1 overexpression increased the level of γ-H2AX (Fig. 5e).

Our results (Figs. 2, 3 and 4) had indicated that arsenic sulfide induction of DSBs was mediated by NFATc3. We therefore hypothesized that arsenic sulfide could also upregulate RAG1 expression. We examined RAG1 levels after arsenic sulfide treatment and found that they were significantly higher than in the control group (Fig. 5f, g, Additional file 1: Figure S5b). To investigate whether RAG1 mediated the NFATc3-silencing effect, we constructed two RAG1-silencing shRNA sequences (shRAG1–1 and shRAG1–2) and infected AGS cells with them individually. We found that when NFATc3 alone was silenced, γ-H2AX was clearly upregulated, while when RAG1 alone was silenced, γ-H2AX was slightly downregulated. However, when NFATc3 and RAG1 were both silenced, there was no upregulation of γ-H2AX (Fig. 5h). These results indicate that arsenic sulfide and NFATc3 cause DSBs through upregulation of RAG1.

### Tumor expression of NFATc3 and RAG1 correlate with survival

**Fig. 6:**
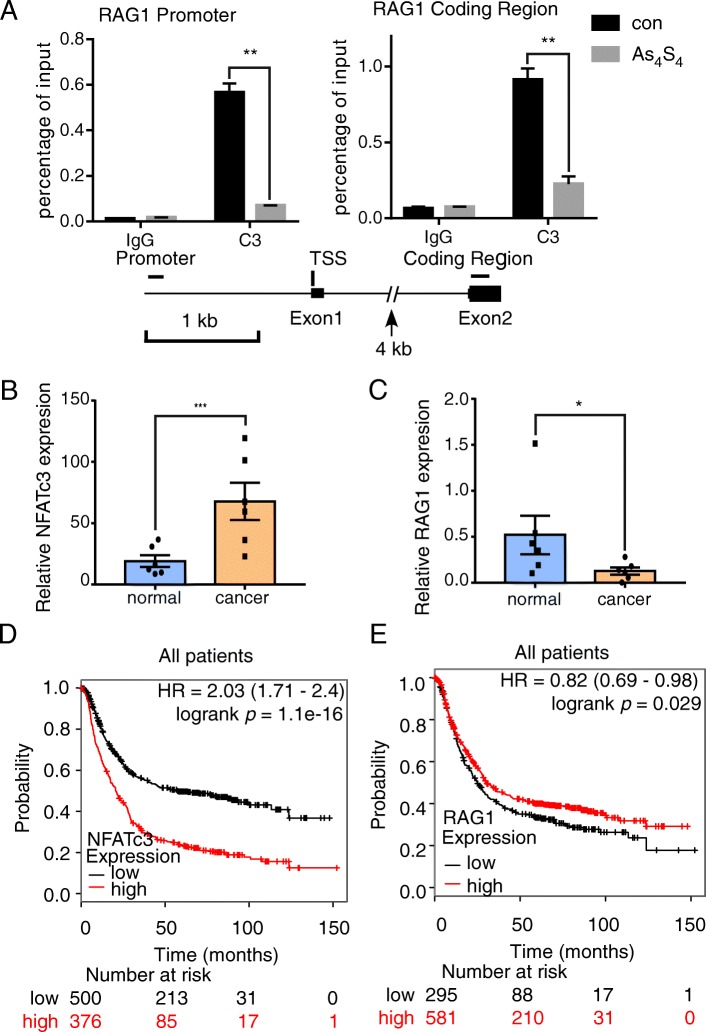
Tumor expression of NFATc3 and RAG1 correlated with survival. **a** ChIP analyses at promoter and coding region of the RAG1 locus in AGS cells upon arsenic sulfide treatment. Error bars reflect mean ± SD calculated from three independent experiments. Statistical significance was assessed using two-tailed Student’s t-test. **p < 0.01. **b, c** qRT-PCR data for NFATc3(**b**) and RAG1(**c**) expression in 6 paired gastric cancer and matched adjacent nonmalignant tissues. Statistical significance was assessed using two-tailed Student’s t-test. *p < 0.05, ***p < 0.001. **d, e** Kaplan–Meier survival curve analysis of the overall survival durations of two groups of gastric cancer patients defined as low or high NFATc3 (**d**) or RAG1 (**e**) expression

The presence of NFAT-binding consensus sites (Additional file 1: Figure S6a) in the promoters of RAG1 (Additional file 1: Figure S6b, c) and the recent description of NFATc1 binding to RAG1 in hair follicle stem cells [26] pointed to RAG1 as a direct target of NFATc3. Using chromatin immunoprecipitation assays in AGS cells (Fig. 6a), we detected binding of NFATc3 to the promoter and exon 2 of the RAG1 gene. These regions contain putative NFATc3 binding sites, suggesting that RAG1 expression was directly regulated by NFATc3. We noted that NFATc3 levels decreased upon arsenic sulfide treatment (Fig. 3a), and this decrease was also associated with decreased NFATc3 occupancy at the RAG1 promoter (Fig. 6a). As a positive control, NFATc3 binding was also detected in the promoter of the IL2 gene in AGS cells (Additional file 1: Figure S6d, e). These results suggested that RAG1 expression was directly regulated by NFATc3. We then further verified NFATc3 and RAG1 dysregulation in six pairs of gastric cancer tissues and matched adjacent nonmalignant tissues by qRT-PCR. The results showed that NFATc3 expression was significantly increased in the gastric cancer tissues (Fig. 6b) while RAG1 expression was significantly decreased in the gastric cancer tissues (Fig. 6c).

To validate our clinical findings in an independent dataset, NFATc3 and RAG1 expression were analyzed using the gastric cancer dataset at www.kmplot.com. The desired Affymetrix ID for NFATc3 was 210556_at and for RAG1 was 206591_at. The survival curve for overall survival (OS) was plotted for 876 patients from the dataset (excluding GSE62254). Patients with NFATc3 upregulation had significantly worse OS compared to those with NFATc3 downregulation (*p* = 1.1e-16, HR = 1.71–2.4; Fig. 6d). Interestingly, low RAG1 expression correlated with poor survival of gastric cancer patients (*p* = 0.029, HR = 0.69–0.98; Fig. 6e). These clinical data support the conclusions that NFATc3 in gastric cancer is over-expressed and that NFATc3 upregulation and RAG1 downregulation are significantly associated with poor prognosis. Furthermore, arsenic sulfide, which enhances ROS targeting NFATc3, may be an effective drug for the treatment of gastric cancer.

### In vivo study of arsenic sulfide in gastric cancer cells

**Fig. 7:**
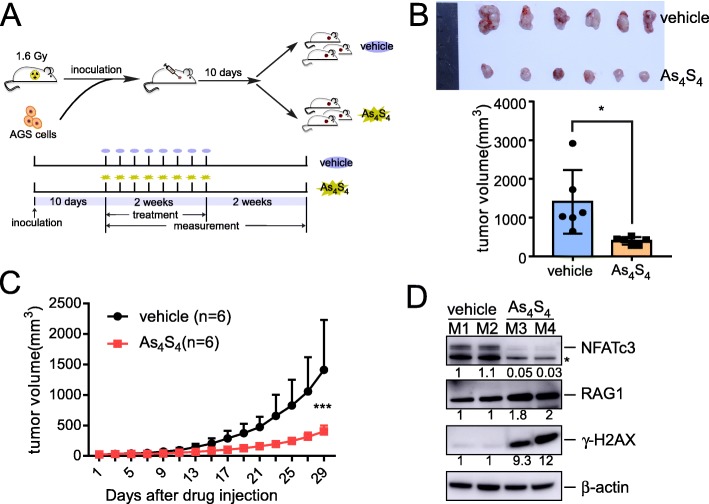
Effect of arsenic sulfide on tumor growth in the xenograft model. **a** Schematic of medication administration in mice injected with AGS cells. **b** Excised tumors and tumor volume in different groups are shown. Statistical significance was assessed using two-tailed Student’s t-test. *p < 0.05. **c** Growth curve showing the changes in the tumor volume in mice after different treatments. Statistical significance was assessed using two-tailed Student’s t-test. ***p < 0.001. **d** Immunoblot analysis of indicated protein expression in excised tumors. Fold changes relative to first line are indicated.

To further evaluate the effects of arsenic sulfide on AGS cells in vivo, an AGS xenograft model was generated by subcutaneous injection of the cancer cells into mice. The mice were randomly divided into two groups, and 10 days after injection, the mice were administered vehicle or 2 mg/kg arsenic sulfide every 2 days for 2 weeks (Fig. 7a). We found that the tumor size in the group treated with arsenic sulfide was significantly reduced compared to the control group (*p* < 0.05, Fig. 7b). Growth curves also showed that the tumor growth rate was significantly reduced after arsenic sulfide treatment (Fig. 7c). Analysis of tumor lysates from control(M1, M2) and arsenic sulfide-treated(M3, M4) mice showed an obvious decrease in the expression of NFATc3 proteins (Fig. 7d), and there was a significant increase in RAG1 and γ-H2AX protein levels in the tumors from mice treated with arsenic sulfide (Fig. 7d).

## Discussion

Gastric cancer is an important health problem as the fourth most common cancer and the second leading cause of cancer deaths worldwide. It is the main contributor to the global burden of disability-adjusted life-years from cancer in men and accounts for 20% of the total burden worldwide, following lung and liver cancers which account for 23 and 28% [27], respectively. We have shown that arsenic sulfide as well as knockdown of NFATc3 induced DSB and RAG1 expression. Forced expression of NFATc3 blocked the induction of DSB and RAG1 expression induced by arsenic sulfide. NFATc3 targeted the promoter of RAG1 for transcriptional inhibition. In addition, we showed that arsenic sulfide stimulated radical species in gastric cancer cells. Mitigation of ROS with the generic antioxidant NAC partially neutralized the cytotoxic activity of arsenic. These results support a role for NFAT in the progress of solid tumors and provide novel insights into the mechanism of arsenic compounds in the solid tumors.

We had previously shown that NFATc3 knockdown impaired colony formation of HCT116 cells and suppressed tumor growth in mice [28]. In the present study, we demonstrate that NFATc3 expression was required for the growth and survival of gastric cancer cells. NFATc3 silencing caused significant growth inhibition and the arrest of cells in the G0–G1 phase. More importantly, the expression of an NFATc3 cDNA carrying synonymous point mutations on the shRNA target sequence rescued the impaired proliferation of cancer cells caused by NFATc3 silencing. Taken together, these data suggest that NFATc3 is required for the growth and survival of gastric cancer cells. In other words, gastric cancer cells may be addicted to NFATc3. Thus, NFATc3 may be a potential target for gastric cancer treatment.

Oncogene addiction enables targeted therapies to affect clinical responses with simultaneously little toxicity. Targeted cancer therapies which interrupt oncogenic molecular pathways driven by mutations, overexpression or translocation of specific genes, such as trastuzumab, an antibody against HER2 (also known as ERBB2) and the VEGFR-2 antibody ramucirumab, have been successfully employed in gastric cancer treatment [29]. Molecularly targeted therapies are most effective in patients who have a specific biomarker in their tumor cells, which indicates the presence of a specific variation that makes the tumor cells susceptible to the targeted agent [30]. Here, we found that NFATc3 is required for the growth and survival of gastric cancer cells. More importantly, arsenic compounds can target NFATc3. Arsenic can not only inhibit the transcription of NFATc3, but also promote the degradation of its protein. At the same time, we also found that arsenic can inhibit the transcriptional activity of NFATc3 by increasing the level of ROS in cells. Arsenic can inhibit the action of NFAT at multiple levels. This makes it an ideal targeted therapy for NFATc3-dependent gastric cancer.

Based on the above preclinical findings, we have applied for a clinical study entitled “Realgar-Indigo Naturalis Formula (RIF) (ingredients: realgar, Indigo naturalis and Salvia miltiorrhiza) combined with irinotecan versus placebo plus irinotecan in the treatment of advanced gastric cancer after first-line treatment: A randomized, double-blind clinical study of the efficacy and safety of patients with locally advanced gastric cancer (Clinical Research No. ChiCTR-INR-16009947). This study will observe the efficacy and safety of realgar compound in the treatment of gastric cancer. The relationship between the expression level of NFATc3 and the clinical efficacy of realgar will be further studied.

The RAG1 and RAG2 proteins form an endonuclease with a preferred DNA substrate, the recombination signal sequence, to initiate the V(D) J recombination process. RAG1 serves as the workhorse for DNA cleavage. RAG1 and RAG2 introduce DSBs between the two participating gene segments and their flanking recombination signal sequences. The prior analysis of RAG1 binding at non-antigen receptor genes was consistent with the model that RAG1 association with active chromatin was restricted to regions containing strong RSSs [31]. This provided an appealing mechanism to limit the genotoxic threat of RAG. However, recently research has revealed that antigen receptor genes are not exceptional in their ability to recruit RAG1 [32]. Open chromatin allows for access to DNA, and RAG1 has substantial non-specific DNA binding activity [33, 34]. Our data suggest that low RAG1 expression correlates with poor survival of gastric cancer patients. NFATc3 silencing abolished the transcriptional suppression of RAG1 leading to increased DNA DSBs in gastric cancer cells. More importantly, arsenic sulfide induces DNA damage leading to cell death by inhibiting NFATc3 in gastric cancer cells. DSBs activate ATM which in turns amplifies and channels the signal by activating downstream kinase CHK-2, that phosphorylates its target protein p53, inducing p21 expression to arrest cell cycle.

## Conclusion

**Fig. 8:**
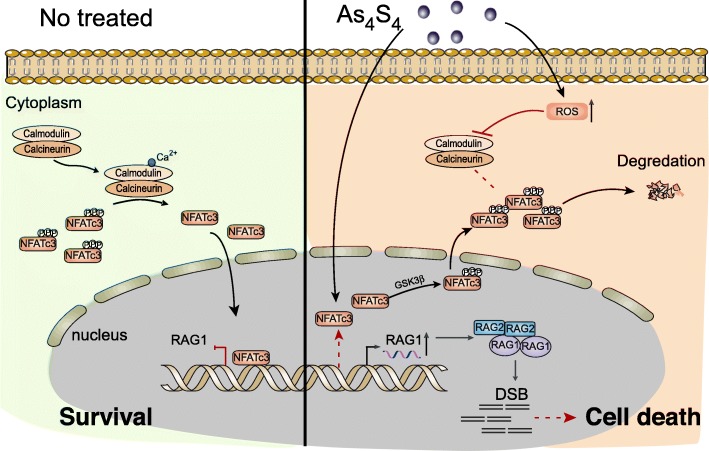
Schematic figure for present study. In untreated cancer cells (left), NFATc3 is dephosphorylated by activated calcineurin, which leads to its nuclear translocation and inhibition of NFAT-mediated RAG1 gene transcription. When arsenic is present (right), NFATc3 is inhibited at the transcriptional level, while increased intracellular ROS levels lead to NFATc3 redistribution and protein degradation. Decreased NFATc3 results in upregulation of RAG1 expression, producing fatal DNA damage

We have shown that RAG1 is a direct target of NFATc3 and that increased expression of NFATc3 in gastric cancer cells suppresses the expression of RAG1 to prevent cell death, while arsenic sulfide inhibits NFATc3 through ROS to induce the expression of RAG1 and increase DSB, leading to increased cell death (Fig. 8). Our data for the first time establishes a link between arsenic sulfide and the NFATc3 pathway to the RAG1 pathway.

## Supplementary information

**Additional file 1: Figure S1.** NFATc3 silencing reduces viability and proliferation of gastric cancer cell line and the shNFATc3-resistant NFATc3 cDNA. **Figure S2.** NFATc3 silencing upregulated DNA damage related genes in MGC803 and MKN45 cells. **Figure S3.** Arsenic sulfide plays a role in inhibiting tumors through NFATc3. **Figure S4.** Arsenic sulfide increase celluar ROS in MGC803 and MKN45 cellsn and CsA redistributing NFATc3 localization. **Figure S5.** NFATc3 silencing alters the expression of RAG1 gene. **Figure S6.** NFATc3 consensus elements in the promoters of Il-2 and RAG1. **Table S1.**Primer list.

## Data Availability

The dataset used and/or analyzed during the current study are available from the corresponding author on reasonable request.

## Abbreviations

CsA: Cyclosporine A
DSB: Double strand-break
NAC: N-acetyl-L-cysteine
NFATc3: Nuclear factor of activated T-cells, cytoplasmic 3
PML: Promyelocytic leukemia protein
RAG1: V(D) J recombination-activating protein 1
ROS: Reactive oxygen species
