# Supplementary material for: Arsenic sulfide induces RAG1-dependent DNA damage for cell killing by inhibiting NFATc3 in gastric cancer cells
Source: J Exp Clin Cancer Res. 2019 Dec 10;38:487. doi: 10.1186/s13046-019-1471-x (PMC6902349; doi:10.1186/s13046-019-1471-x)
Supplement: Supplementary file 1 — Additional file 1: Figure S1. NFATc3 silencing reduces viability and proliferation of gastric cancer cell line and the shNFATc3-resistant NFATc3 cDNA. Figure S2. NFATc3 silencing upregulated DNA damage related genes in MGC803 and MKN45 cells. Figure S3. Arsenic sulfide plays a role in inhibiting tumors through NFATc3. Figure S4. Arsenic sulfide increase celluar ROS in MGC803 and MKN45 cellsn and CsA redistributing NFATc3 localization. Figure S5. NFATc3 silencing alters the expression of RAG1 gene. Figure S6. NFATc3 consensus elements in the promoters of Il-2 and RAG1. Table S1.Primer list. [file 13046_2019_1471_MOESM1_ESM.pdf]

**Figure S1. NFATc3 silencing reduces viability and proliferation of gastric cancer cell line and the shNFATc3-resistant NFATc3 cDNA.**

**(a-d)** NFATc3 silencing induced G1/S cell cycle arrest in MKN45 (a, b) and MKC803 (c, d). Cells were infected with lentivirus shC3-1 or shScr analyzed by flow cytometry after infection day1 to day5. The percentage of cell population at G1, S, and G2/M phases are represented as mean ± S.D. of three independent experiments. **(e)** Target sequence for the NFATc3-shRNA (upper case) and mutated nucleotides (red) introduced in the target sequence without changing the amino acid sequence of NFATc3.

**Figure S2. NFATc3 silencing upregulated DNA damage related genes in MGC803 and MKN45 cells.**

**(a-b)** mRNA levels analyzed by q-RT-PCR of NFATc3 (a) and p21 (b) in shC3-1 or shScr infected MKN45 cells after infection day1 to day3. Statistical significance was assessed using two-tailed Student's t-test. *$p < 0.05$; ***$p < 0.001$. **(c)** MGC803 cells were infected with lentivirus shC3-1 or shScr analyzed after infection day1 to day3. Immunoblot of NFATc3 and p-CHK2/CHK2 and γ-H2AX expression in that infected cells. Fold changes relative to shScr are indicated.

**Figure S3. Arsenic sulfide plays a role in inhibiting tumors through NFATc3.**

**(a)** qPCR analysis of NFATc3 expression in arsenic sulfide treated AGS cells. Statistical significance was assessed using two-tailed Student's t-est. **$p < 0.01$; ***$p < 0.001$. **(b)** qPCR analysis of NFATc3 expression in arsenic sulfide treated AGS cells. Statistical significance was assessed using two-tailed Student's t-test. **$p < 0.01$; ***$p < 0.001$. **(c, d)** Arsenic sulfide

treatment induced G1/S cell cycle arrest in MKN45(c). The percentage of cell population at G1, S, and G2/M phases are represented as mean ± S.D. of three independent experiments(d).

**Figure S4. Arsenic sulfide increase celluar ROS in MGC803 and MKN45 cells and CsA redistributing NFATc3 localization.**

**(a, b)** MGC803 (a) and MKN45 (b) treated with or without arsenic sulfide were stained with DCFH-DA (10μM, 20min, 37℃) and MFI of DCFH-DA was analyzed in each cell subset. Statistical significance was assessed using two-tailed Student’s t-test. *$p < 0.05$; **$p < 0.01$; ***$p < 0.001$. **(c)** The distribution of NFATc3 in cytoplasm and nucleus after CsA treatment. Fold changes of NFATc3 protein relative to first line are indicated. **(d)** Relative survival varbility of AGS cells treated with contral, arsenic sulfide(5uM), NAC (10mM) and arsenic sulfide plus NAC measured by MTT assay. Statistical significance was assessed using two-tailed Student’s t-test. *$p < 0.05$.

**Figure S5. NFATc3 silencing alters the expression of RAG1 gene.**

**(a)** qPCR analysis of RAG1 expression in indicated MKN45 cells. Statistical significance was assessed using two-tailed Student’s t-test. *$p < 0.05$; **$p < 0.01$; ***$p < 0.001$. **(b)** qPCR analysis of RAG1 expression in arsenic sulfide treated MGC803 cells. Statistical significance was assessed using two-tailed Student’s t-test. ***$p < 0.001$.

**Figure S6. NFATc3 consensus elements in the promoters of Il-2 and RAG1**

**(a)** The presence of NFAT-binding consensus sites in the promoters of IL2. ( swissregulon.unibas.ch/sr/). **(b, c)** Promoter (b) and coding region (c) sequences of human *RAG1* gene contain NFAT consensus elements (boxes). Primer used for ChIP-qPCR and the start codon

are indicated (underlined). **(d)** ChIP analyses at promoter of the IL2 locus on the indicated cells as positive control.**(e)** Promoter sequences of human *IL2* gene contain NFAT consensus elements (boxes). Primer used for ChIP-qPCR and the start codon are indicated (underlined).

Table S1.primer list

| primer | sequence |
|---|---|
| HUMAN-NFATc3-qPCR-F | CGAGGGGCAGTAAAAGCATC |
| HUMAN-NFATc3-qPCR-R | CAGTGATTCGATGCACCTGG |
| HUMAN-GAPDH-qPCR-F | GGCACAGTCAAGGCTGAGAATG |
| HUMAN-GAPDH-qPCR-R | ATGGTGGTGAAGACGCCAGTA |
| HUMAN-p21-qPCR-F | GTCTTGTACCCTTGTGCCTC |
| HUMAN-p21-qPCR-R | GGTAGAAATCTGTCATGCTGG |
| HUMAN-RAG1-qPCR-F | CTGTTCCGGGTGAGATCCTTT |
| HUMAN-RAG1-qPCR-R | TAACAATGGCTGAGTTGGGAC |

# Figure S1

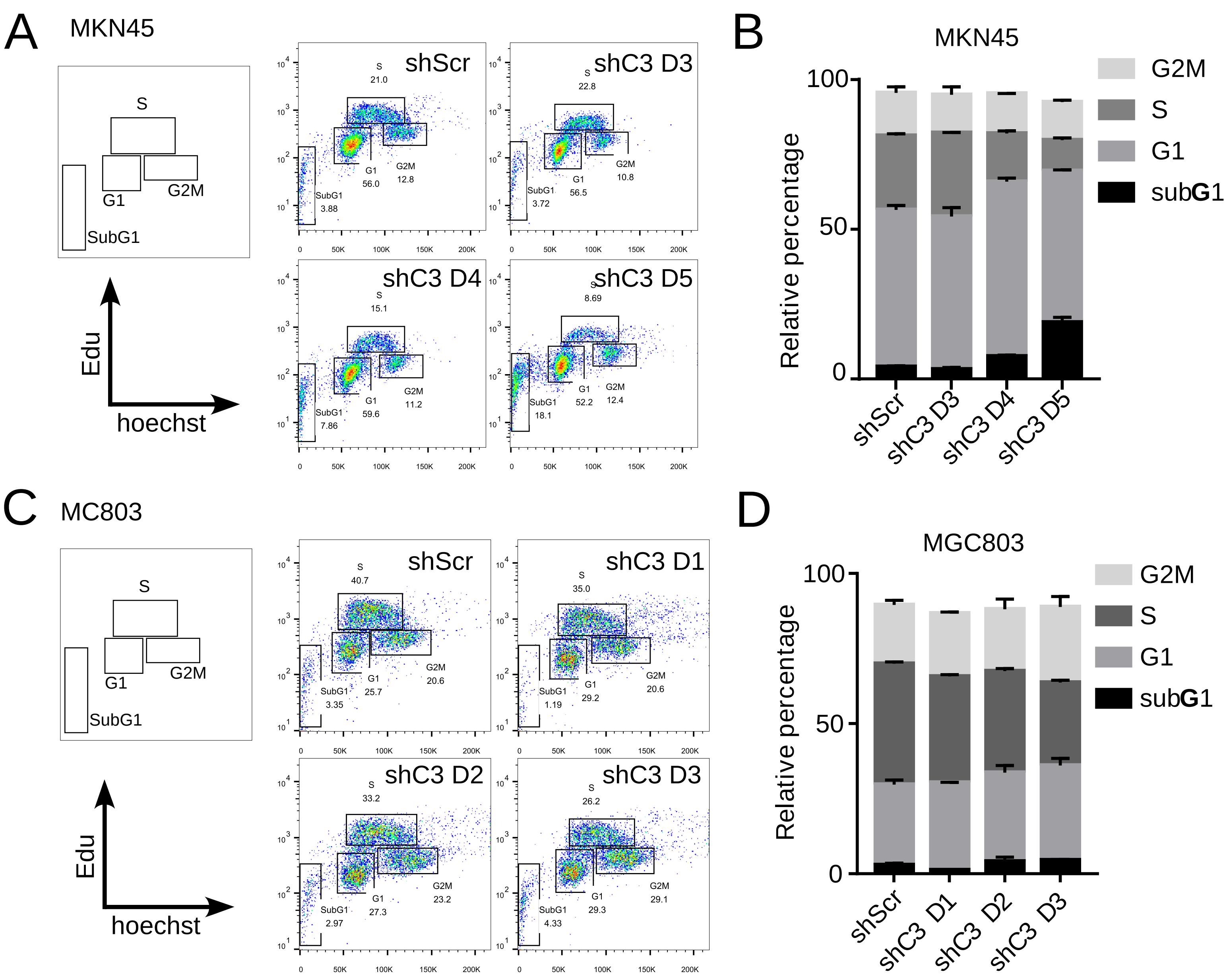


E

907-913aa shC3-1-target

NFATc3 TCG TCT CAG TTA CAA CCT ATT A

NFATc3m TCC TCA CAA CTT CAG CCC ATC A

Ser Ser Gln Leu Gln Pro Ile

# Figure S2

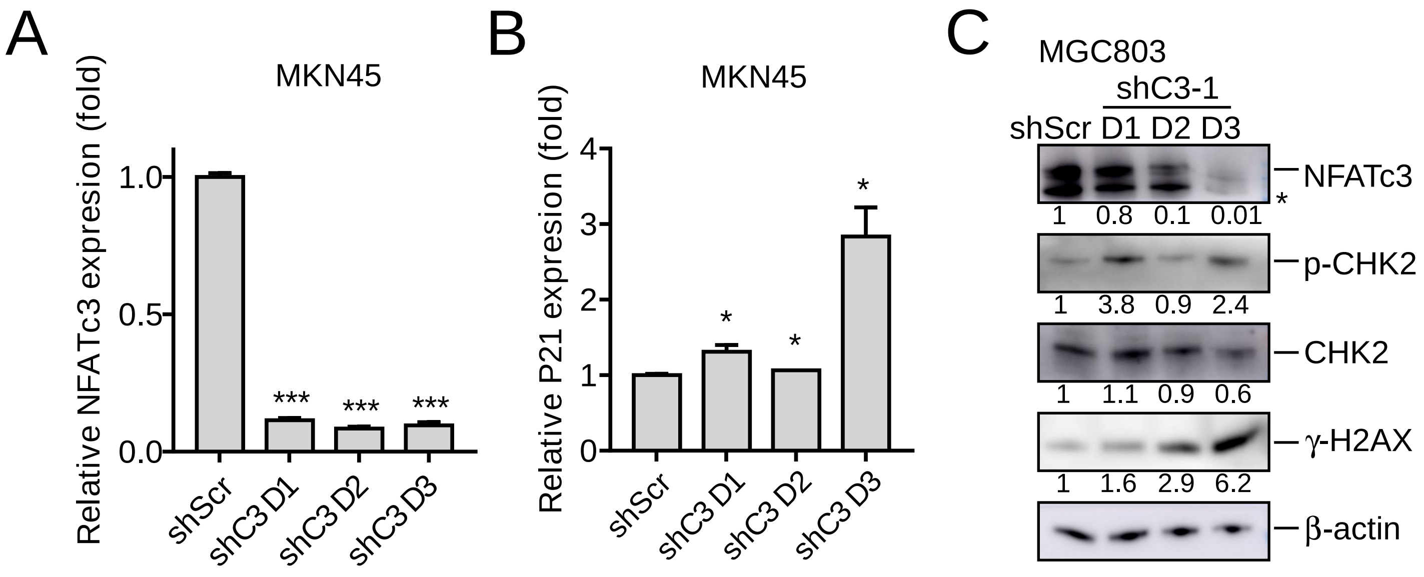
A
MKN45
Relative NFATc3 expresion (fold)
1.0
0.5
0.0
***
***
***
shScr
shC3 D1
shC3 D2
shC3 D3
B
MKN45
Relative P21 expresion (fold)
4
3
2
1
0
*
*
*
shScr
shC3 D1
shC3 D2
shC3 D3
C
MGC803
shC3-1
shScr D1 D2 D3
NFATc3
*
1 0.8 0.1 0.01
p-CHK2
1 3.8 0.9 2.4
CHK2
1 1.1 0.9 0.6
γ-H2AX
1 1.6 2.9 6.2
β-actin

# Figure S3

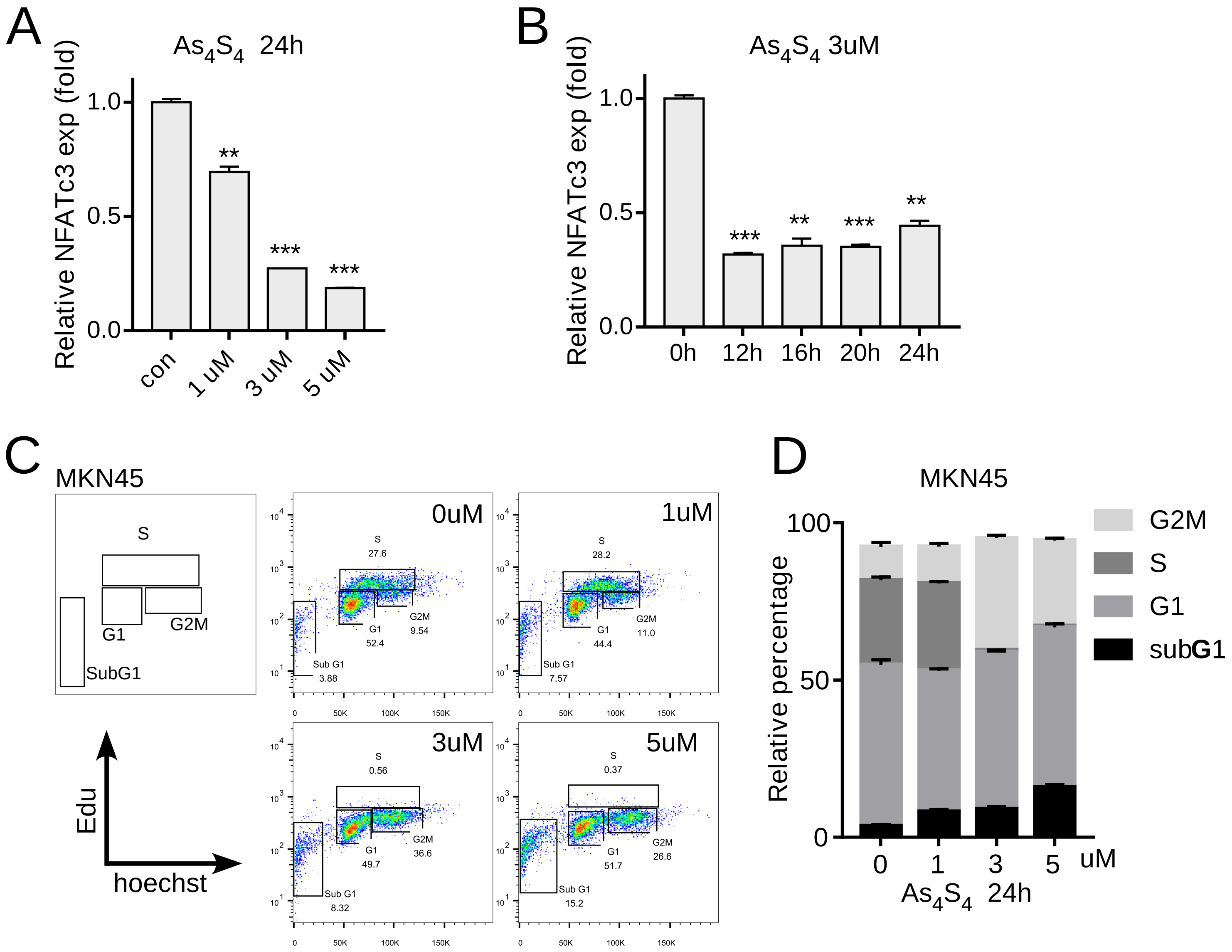
A
As4S4 24h
Relative NFATc3 exp (fold)
1.0
0.5
0.0
**
***
***
con
1 uM
3 uM
5 uM
B
As4S4 3uM
Relative NFATc3 exp (fold)
1.0
0.5
0.0
***
**
***
**
0h
12h
16h
20h
24h
C
MKN45
S
G1
G2M
SubG1
Edu
hoechst
0uM
S
27.6
G1
52.4
G2M
9.54
Sub G1
3.88
1uM
S
28.2
G1
44.4
G2M
11.0
Sub G1
7.57
3uM
S
0.56
G1
49.7
G2M
36.6
Sub G1
8.32
5uM
S
0.37
G1
51.7
G2M
26.6
Sub G1
15.2
0
50K
100K
150K
D
MKN45
Relative percentage
100
50
0
G2M
S
G1
subG1
0
1
3
5
uM
As4S4 24h

# Figure S4

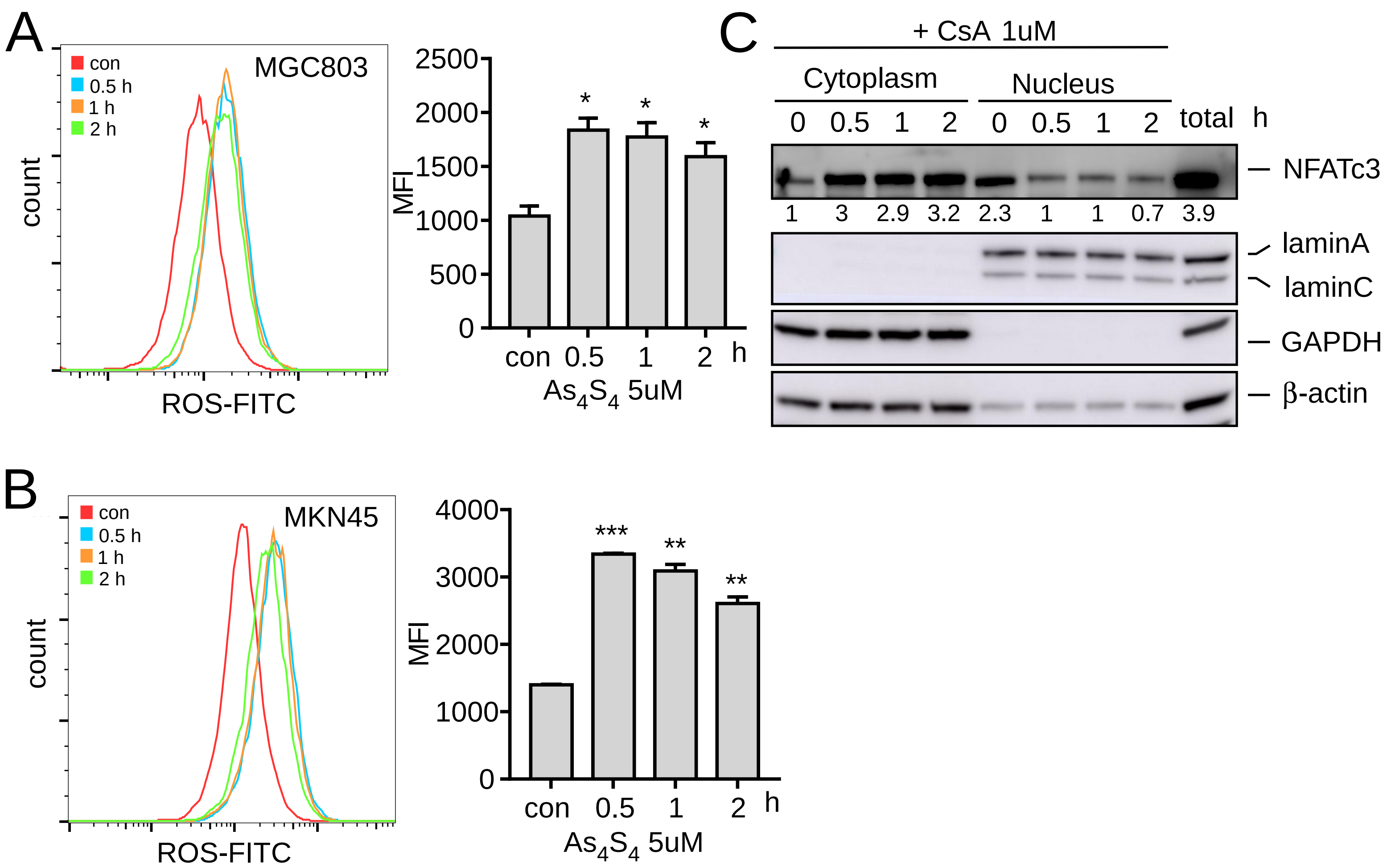

A
con
0.5 h
1 h
2 h
MGC803
count
ROS-FITC
2500
2000
1500
1000
500
0
MFI
*
*
*
con
0.5
1
2
h
As4S4 5uM
C
+ CsA 1uM
Cytoplasm
Nucleus
0
0.5
1
2
0
0.5
1
2
total
h
NFATc3
1
3
2.9
3.2
2.3
1
1
0.7
3.9
laminA
laminC
GAPDH
β-actin
B
con
0.5 h
1 h
2 h
MKN45
count
ROS-FITC
4000
3000
2000
1000
0
MFI
***
**
**
con
0.5
1
2
h
As4S4 5uM

# Figure S5

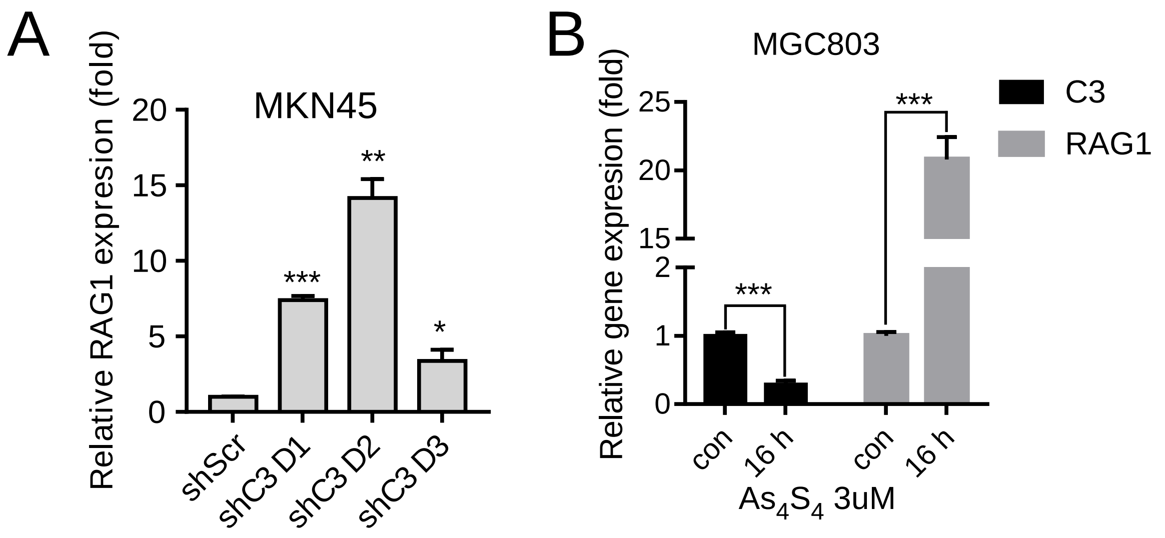
A
MKN45
Relative RAG1 expresion (fold)
20
15
10
5
0
***
**
*
shScr
shC3 D1
shC3 D2
shC3 D3
B
MGC803
Relative gene expresion (fold)
25
20
15
2
1
0
***
***
C3
RAG1
con
16 h
con
16 h
As4S4 3uM

# Figure S6

A

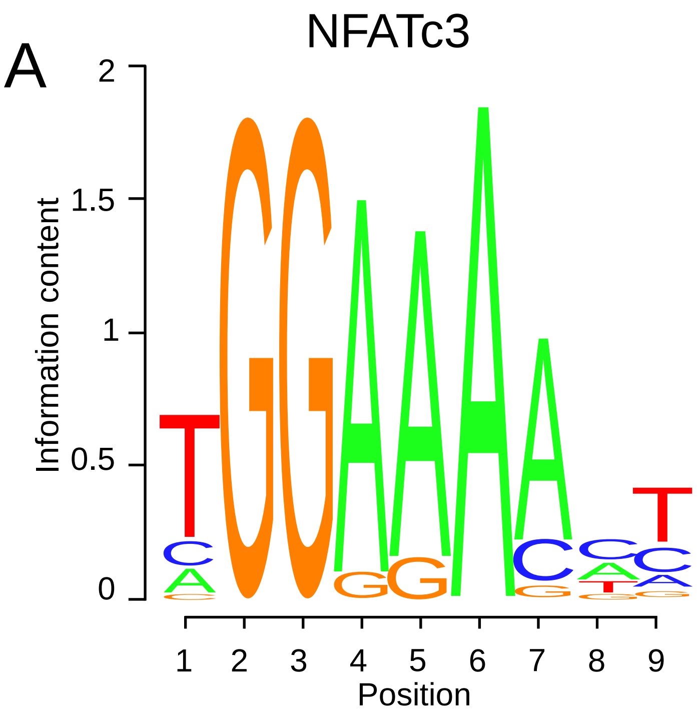


D

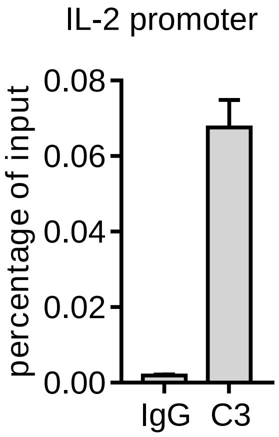


B

aattccatagtgttgttaatatctgatgaacatctacaaagaggcagacatcgtttcaagtatttcctataataactgt
cattgtataacattattgactctttgaggtatatactactagcctttcctttttacagatgtggaactgaggcacagaga
tatagaaccctggcaaaagctggggcttatactgactgacaagaccatttccaacccaaa

C

GTACCTCAGCCAGCATGGCAGCCTCTTTCCCACCCACCTTGGGACTCAGTTCTGCCCCAG
ATGAAATTCAGCACCCACATATTAAATTTTCAGAATGGAAATTTAAGCTGTTCCGGGTGAGAT
CCTTTGAAAAGACACCTGAAGAAGCTCAAAAGGAAAAGAAGGATTCCTTTGAGGGGAAAC
CCTCTCTGGAGCAATCTCCAGCAGTCCTGGA

E

acatgttcagtgtagttttatgacaaagaaaattttctgagttacttttgtatccccacccccttaaagaaaggaggaaa
aactgtttcatacagaaggcgttaattgcatgaattagagctatcacctaagtgtgggctaatgtaacaaagagggat
ttcacctacatccattcagtcagtctttggggggtttaaagaaattccaaagagtcatcagaa
